# Present and future treatment strategies for coronavirus disease 2019

**DOI:** 10.1186/s43094-021-00238-y

**Published:** 2021-04-09

**Authors:** Engy Elekhnawy, Amal Abo Kamar, Fatma Sonbol

**Affiliations:** Pharmaceutical Microbiology, Faculty of Pharmacy, Tanta University, Tanta, El Gharbia Governorate Egypt

**Keywords:** COVID-19, SARS-Cov-2, Cytokine storm, Respiratory distress syndrome

## Abstract

**Background:**

The recent pandemic of coronavirus disease 2019 (COVID-19) has resulted in many challenges to the healthcare organizations around the world. Unfortunately, until now, there are no proven effective therapeutic agents against this virus.

**Main body:**

Several evolving studies suggest repurposing a potential list of drugs which have appropriate pharmacological and therapeutic effects to be used in treating COVID-19 cases. In the present review, we will summarize the potential drugs suggested to be repurposed to be utilized in the treatment of COVID-19 patients like lopinavir-ritonavir, ribavirin, baloxavir marboxil, favipiravir, remdesvir, umifenovir, chloroquine, hydroxychloroquine, azithromycin, corticosteroids, losartan, statins, interferons, nitric oxide, epoprostenol, tocilizumab, siltuximab, sarilumab anakinra, and ruxolitinib. In addition, we discussed the possible future therapeutic regimens based on the recent molecular and genomic discoveries.

**Conclusion:**

This review could provide beneficial information about the potential current and future treatment strategies to treat the pandemic COVID-19 disease.

## Background

In the past 20 years, many viral epidemics like the severe acute respiratory syndrome coronavirus (SARS-CoV) in 2002, H_1_N_1_ influenza virus in 2009, and the Middle East respiratory syndrome coronavirus (MERS-CoV) in 2012 have been documented. In December 2019, an epidemic of cases having unexplained low respiratory tract infections detected in Wuhan, China, was first reported. The etiology of this disease was then attributed to a new virus that belongs to the coronavirus (CoV) family called coronavirus disease 2019 (COVID-19). It is also termed SARS-CoV-2 as it is very similar to SARS-CoV. This novel virus is very contagious and it has a very quick spread worldwide [1]. In 2020, the WHO declared COVID-19 as a pandemic disease. From December 2019 to July 2020, more than 10.4 million of COVID-19 cases have been recorded in more than 188 countries, resulting in more than 510,000 [1].

The CoVs are positive-stranded RNA viruses isolated from different animal species. They can be transmitted to humans where they can cause illness that range from common cold to more serious diseases like MERS and SARS. They belong to the *Coronaviridae* family, which is the largest family in the order *Nidovirales*. Family *Coronaviridae* comprises two subfamilies: subfamily *Orthocoronavirinae* and subfamily *Torovirinae*. The subfamily *Orthocoronavirinae* includes four genera: alphacoronavirus, betacoronavirus, gammacoronavirus, and deltacoronavirus. The viruses SARS-CoV, MERS-CoV, and COVID-19 are betacoronaviruses [2].

Coronaviruses are spherical and they are characterized by the presence of spike proteins that project from the viral surface. Due to the appearance of the viral particle under the electron microscope as a royal crown, it was named coronavirus from the Latin word corona meaning crown. CoVs are enveloped viruses and they have some structural proteins like spike (S), membrane (M), envelope (E), hemagglutinin-esterase (HE), and nucleocapsid (N) proteins. The S, M, and E, HE proteins are embedded in the envelope of the virus [1]. Nevertheless, the N protein interacts with the genetic material of the virus (RNA) forming the nucleocapsid in the core of the virus which is essential for packaging of the viral RNA into the viral particle during the viral assembly. The spike protein (S protein) is a glycosylated protein forming spikes on the surface of the virus and it mediates the entry of the virus into the cells of the host. The membrane protein (M protein) gives the virus its shape and is important together with the E protein in forming the mature envelope of the virus. The E protein also has a role in the release of the viral particles from the host cells. The HE protein is a glycoprotein which helps the virus in the attachment to the host cell surface; also it has acetyl-esterase activity [1].

There are a number of challenges in the treatment and prevention of COVID-19 that contributes to the high threat of the disease. They are summarized below. A prior good understanding of these difficulties is essential in strategizing new therapeutic alternatives.

Similar to all RNA genomes, the COVID-19 genome lacks the proofreading mechanism and so it mutates frequently. Mutations can offer the virus certain selective advantages, for instance resistance to the currently developed vaccines and antiviral drugs. In addition, mutation enables the virus to escape from the adaptive immunity of the host and increases its infectivity and virulence. Also, it can lead to greater spread of the virus either horizontally (i.e., from one individual to another within the same species) and/or vertically (i.e., crossing the host species, for instance from bats to man) [3].

A clinically relevant aspect of the pandemic COVID-19 is its ability to induce the so-called cytokine storm (CS) that consists of interleukin-1 (IL-1), interleukin-6 (IL-6), interleukin-8 (IL-8), and tumor necrosis factor-alpha (TNF-α). These pro-inflammatory mediators can provoke systemic inflammatory response syndrome, resulting in acute respiratory distress syndrome (ARDS). The pathological changes that usually occur include diffuse alveolar damage due to immunological injury and viral infection, as well as multi-organ failure including airways destruction, vascular endothelial damage, plasma leakage, and extensive microthrombus formation [4]. Pneumonia that frequently occurs in cases with COVID-19 either results as a direct consequence of the viral infection in the lung or arises due to secondary bacterial infections after the viral episode [5].

COVID-19 can be deadly to particular groups in the population, like the elderly and individuals having immune deficiency. Thus, this group is in the highest need of prophylaxis or more intensive treatments against COVID-19 [6].

The brief background discussed above clearly showed that the reliable prevention and treatment of COVID-19 represent a very critical public health need. In this review, we started with summarizing the current treatment for COVID-19. We then presented and critically reviewed the prospective future treatments for COVID-19 which are at various stages of development.

## Main text

### Current treatments of COVID-19

**Fig. 1:**
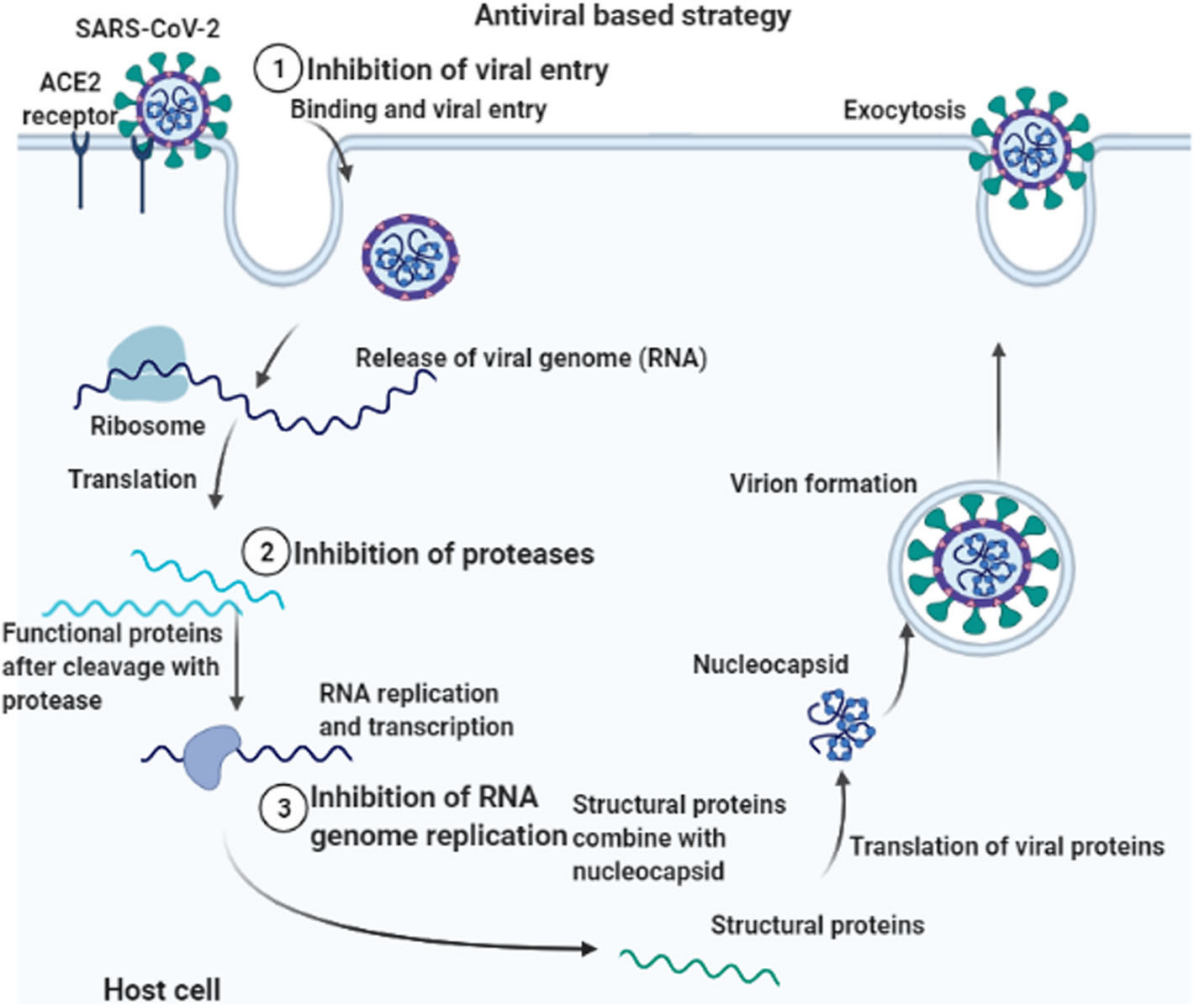
Targets for antiviral drugs currently available treatment for COVID-19 combined with immune modulatory agents and thromboprophylaxis

At the present time, the treatment strategies dealing with COVID-19 infection is mainly supportive, like mechanical ventilation and oxygen supplementation, and the reduction of the viral transmission in the community is the greatest weapon. Although antiviral treatments for COVID-19 have not been approved yet, several approaches have been proposed including repurpose (reposition) some therapeutics approved for other conditions for COVID-19 patients (Fig. 1). The repurposing of the antivirals includes the use of lopinavir-ritonavir, ribavirin, baloxavir marboxil, favipiravir, remdesvir, and umifenovir (arbidol). Other drugs which are not used as antivirals but have potential activity against COVID-19 include the use of chloroquine, hydroxychloroquine, azithromycin, corticosteroids, losartan, statins, interferons, nitric oxide, and epoprostenol which are examples for the repurposing strategy. Also, some agents are proposed to be used in severely ill COVID-19 patients exhibiting cytokine release syndrome (CRS) including tocilizumab, siltuximab, sarilumab anakinra, and ruxolitinib. In addition, thromboprophylaxis is suggested to be applied to all hospitalized patients with COVID-19. Passive transfer of antibodies from convalescent patient sera is another rationale that is currently used.

#### Lopinavir-ritonavir

Lopinavir is a protease inhibitor used against the human immunodeficiency virus (HIV) type 1. It is used in combination with ritonavir in order to increase its plasma half-life by inhibition of the cytochrome P450. It has an in vitro activity against SARS-CoV, MERS-CoV, and COVID-19 [7]. On the other hand, Chen et al. [8] and Cao et al. [9] did not observe any benefits from using lopinavir-ritonavir in hospitalized patients with severe COVID-19. Thus, their potential effectiveness needs to be investigated by further clinical studies.

#### Ribavirin

It is a guanosine analog which interferes with the replication of RNA and DNA viruses. Nevertheless, the antiviral activity of ribavirin is not only limited to its interference with polymerases but also it interferes with RNA capping that is essential to prevent RNA degradation. It presented an in vitro activity against SARS-CoV, MERS-CoV, and COVID-19 [10]. However, Tong and his colleagues [11] have reported that ribavirin did not provide a survival benefit in comparison with the control treatment (involving only supportive therapy). Its in vivo activity against SARS-CoV-2 needs further investigations.

#### Baloxavir marboxil

They are new inhibitors of RNA replication in influenza virus that act by targeting different protein subunits of the influenza polymerase complex. Baloxavir marboxil inhibits cap-dependent endonuclease enzyme which is involved in the initiation of mRNA synthesis [12]. Thus, these drugs are proposed to be used against SARS-CoV-2. Nevertheless, Lou et al. [13] reported that they could not prove a benefit from addition of either baloxavir marboxil in the treatment of COVID-19 patients.

#### Favipiravir

It is a guanine analogue and prodrug which first enters the infected host cells through endocytosis and then it is transformed into active form through phosphorylation. Its antiviral activity is demonstrated through selectively targeting the viral conservative catalytic domain of RNA-dependent RNA polymerase, leading to interruption the process of the nucleotide incorporation during the viral RNA replication. Recent in vitro and human studies have used favipiravir as an experimental agent against COVID-19 [14]. Currently, Chinese researchers have completed clinical studies on favipiravir, and it showed promising clinical efficacy in treatment of patients having COVID-19. Therefore, favipiravir will be included in the future treatment plan owing to the safety, evident efficacy, and availability of the drug [5]. Another study has revealed that patients with COVID-19 who received favipiravir had a lower mean duration of hospitalization and none of them needed mechanical ventilation [15]. On the other hand, a research group in China have not found a benefit from addition of favipiravir to the treatment protocol of COVID-19 patients [13]. They stated that the viral negativity, clinical symptoms, and laboratory tests did not provide any additional benefits to the clinical outcomes after using favipiravir in their clinical study.

#### Remdesvir

Remdesivir is a prodrug of an adenosine analogue having a broad antiviral spectrum. In vitro, remdesivir inhibits all human and animal coronaviruses, including COVID-19, and has shown antiviral and clinical effects in animal models of SARS-CoV and MERS-CoV. Furthermore, remdesivir has already had effective results in the USA in the fight against COVID-19 [16]. In a cohort study conducted by Grein and his colleagues [17] on hospitalized patients with severe COVID-19 disease, they noticed a clinical improvement in 68% of patients after administration of remdesvir. Yet, remdesivir needs more clinical trials to evaluate its effectiveness and safety for COVID-19 patients.

#### Umifenovir (arbidol)

It is an antiviral drug used in treatment of influenza infection. It can interact with the viral hemagglutinin (HA) and thus inhibiting the fusion of the viral particle to the host cell membrane. It is found that this drug inhibited crucial stages of the COVID-19 replication cycle in vitro [7]. In addition, research findings [18], conducted in Iran, showed that arbidol, significantly contributed to both clinical and laboratory improvements in COVID-19 patients.

#### Chloroquine and hydroxychloroquine

Chloroquine and hydroxychloroquine (a less toxic derivative of chloroquine) are antimalarial drugs which are widely used in the treatment of rheumatic diseases and have also presented a promising activity against COVID-19. Interestingly, they inhibited the virus when the tested cells were treated with it before and/or after exposure to COVID-19, which suggests both prophylactic and therapeutic effects of these drugs. They can also affect the entry and replication of COVID-19 [19]. Nevertheless, John et al. [20] reported the potential hazard of chloroquine of induction of unwanted prolongation of QT-interval as it blocks the KCNH2-encoded HERG/Kv11.1 potassium channel leading to sudden cardiac death.

#### Azithromycin

It is bacteriostatic antibiotic that is widely used in treatment of many Gram-positive infections. Secondary bacterial infection pneumonia has been reported in several patients with COVID-19. Thus, azithromycin is important in treatment of pneumonia caused by bacteria [21]. In addition to its antibacterial activity, it has been revealed to have an immunomodulatory and anti-inflammatory effects thus it has a role in the reduction of the complications caused by the respiratory viral infections like SARS-CoV, MERS-CoV, and COVID-19 [22]. However, accumulating evidence advocates that azithromycin could have arrhythmia-related adverse cardiac effects via QT prolongation [23, 24] which could increase the risk of sudden cardiac death [25]. Interestingly, Gautret and his colleagues [26] proved that there is a large benefit from administration of a combination of hydroxychloroquine and azithromycin in 80 patients with COVID-19.

#### Corticosteroids

They are potent anti-inflammatory drugs which may prevent the occurrence of CRS in patients with COVID-19. Current animal experiments provided evidence for the effect of corticosteroids like dexamethasone, hydrocortisone, and methylprednisolone in decreasing the mortality, reduction of inflammation, attenuating the acute lung injury, reduction the period of hospitalization, decreasing the need for ventilation, and improving the survival in the severely ill patients having ARDS with doses of 15 mg/day, 400 mg/day, and 1 mg/kg/day, respectively [27]. When corticosteroids are used early in adult patients with non-critical COVID-19 pneumonia, the clinical outcomes could be worsened [28]. Although many studies were conducted to investigate the efficacy of corticosteroids in treatment of COVID-19 patients, controversy still exists as some studies have shown its benefit [29], while other studies shown no benefit [30] or a suggestion of significant harm in critically ill patients [31, 32]. So, clinical trials are urgently needed to be carried out in this aspect in order to clarify both the advantages and disadvantages of using corticosteroid therapy in patients having COVID-19.

#### Losartan

Losartan is an angiotensin receptor blocker that blocks angiotensin II. There has been considerable controversy over the use of angiotensin receptor blocker (ARBs) like losartan and angiotensin-converting enzyme (ACE) inhibitors (which are mainly used in treatment of elevated blood pressure) in patients with COVID-19 [33]. COVID-19 virus uses the angiotensin-converting enzyme 2 (ACE2) receptors to enter the host cells and subsequently downregulates its expression after infecting the cells leading to unopposed pro-inflammatory effects of angiotensin II [34]. Through blocking of the angiotensin II receptor, it is proposed that the utilization of losartan can lead to upregulation of the ACE2 receptor, and therefore decrease the pulmonary inflammation, fibrosis, and edema leading to a decrease in the rate and severity of the acute lung injury [33]. On the other hand, COVID-19 utilizes angiotensin-converting enzyme 2 (ACE2) as a receptor binding domain for its S protein. Thus, the increased expression of ACE2 may potentially facilitate COVID-19 infections [34]. An in silico study [35] was conducted in order to investigate the probable modulatory effect of losartan in some critical points of SARS-CoV2 replication cycle and it was elucidated that losartan has high affinity to ACE2. Bengtson and his colleagues [36] carried out a study to investigate the safety of losartan in COVID-19 patients and they reported its safety.

#### Statins

They are lipid-lowering drugs which have exhibited anti-inflammatory and immune-modulatory effects. Previous studies suggested the effectiveness of statin therapy in treating hospitalized influenza patients. In addition, statins have proven to have an anti-thrombotic and anticoagulant effects [37] via interference with the coagulation cascade [38] and downregulation of the clot formation by augmentation of thrombomodulin which binds to thrombin leading to activation of protein C and lowering the plasma levels of factors Va and VIIIa [39]. Thus, these findings may be encouraging to advocate statins as an adjuvant therapy for patients with COVID-19 which are highly susceptible to blood clots that could lead to mortality. However, the use of statins in COVID-19 patients needs first more studies to be conducted to ensure its efficacy.

#### Interferon

Type I interferons (IFNs)-α/β are broad spectrum antivirals which can exhibit both direct inhibitory effects on the viral replication and supporting the host immune response in order to clear the viral infection [40]. Recent studies have revealed that treatment with IFNs-α/β significantly decreased the duration of the detectable viruses in the upper respiratory tract as well as reduced blood levels for the inflammatory markers like Il-1, IL-6, and IL-8 [41]. Thus, these findings advocate using IFNs-α/β as a therapeutic strategy in COVID-19 cases; yet, this needs additional investigation.

#### Nitric oxide and epoprostenol

Both inhaled nitric oxide and inhaled epoprostenol (a naturally occurring prostaglandin) have been widely studied pulmonary vasodilator agents which are used as rescue therapy in mechanically ventilated patients with COVID-19, having severe ARDS and hypoxemia [14]. A study was carried out in the ICUs of a large academic medical center in the USA on critically ill COVID-19 patients which revealed that inhaled epoprostenol and inhaled nitric did not produce a significant increase in the oxygenation metrics. However, the study highlighted that administration of inhaled epoprostenol and inhaled nitric oxide could be considered in patients with severe respiratory failure due to COVID-19 [42].

#### Tocilizumab, siltuximab, and sarilumab

They are recombinant humanized monoclonal antibodies which are IL-6 receptor antagonists that block the biological activity of IL-6. They may potentially combat the release of cytokine and pro-inflammatory mediators leading to reduction in the pulmonary symptoms in severely ill patients having COVID-19 [41]. Khan and his colleagues [43] reported that tocilizumab, siltuximab, and sarilumab were associated with a lower relative risk of mortality.

#### Anakinra

It is a recombinant monoclonal antibody which is IL-1 receptor antagonist and is proposed to be used in severely ill patients with COVID-19 to overcome the CRS [44]. A study performed by Kooistra and his colleagues [45] on 21 severely ill COVID-19 patients treated with anakinra and they compared the clinical outcomes with a group of standard care. They observed that anakinra was effective in reduction of the clinical signs of hyperinflammation. In addition, another research group in Italy [46] reported that anakinra was effective in the management of a critical case of COVID-19.

#### Ruxolitinib

It is a selective inhibitor of Janus kinases (JAK) 1 and 2 which are tyrosine kinases in the host cell cytoplasm. They link cytokine signaling from the membrane receptors. As several patients with COVID-19 having severe respiratory disease due to CRS, it is hypothesized that JAK-inhibitors might have a beneficial role in treating such patients [47]. D'Alessio et al. [48] conducted a study on the effectiveness of ruxolitinib in COVID-19 patients and found that it significantly reduced the mortality without any adverse effect in the treated patients compared to controls.

#### Thromboprophylaxis

Individuals who are hospitalized with COVID-19 are frequently having serious respiratory failure and have elevated serum levels of D-dimer which is an initial screening indicator for venous thromboembolism (VTE). So thromboprophylaxis (prophylactic anticoagulation with anticoagulants, for instance, heparin) is an important part in the management of the critically ill patients having COVID-19 [49]. Anticoagulation Forum and American College of Cardiology recommend continuous monitoring of D-dimer, platelet count, PTT, and fibrinogen levels during administration of anticoagulants in COVID-19 patients. In addition, they recommend at least an anticoagulation course of 3 months for patients who started the anticoagulation therapy for a presumed provoked thrombus from the inflammatory state of COVID-19 disease [50].

### Passive antibody transfer from convalescent patient plasma

Convalescent plasma (CP) therapy involves a recovery of blood plasma (containing neutralizing antibodies against a certain virus) from persons who have recuperated from an infection, and its administration to infected patients to improve the clinical outcomes. Patients with resolved viral infection will have high titer of polyclonal antibodies to different viral antigens of SARS-CoV-2 which will neutralize the virus [51]. Some studies were conducted by several researchers in different parts of the world on the role of CP transfusion in treatment of COVID-19 patients. Duan et al. [52] noted disappearance of viremia in 7 days and the clinical symptoms rapidly improved within 3 days after CP transfusion by severely ill patients. In addition, other researchers reported that CP transfusion for COVID-19 patients was effective and safe [53, 54]. Unfortunately, the exponential growth of the outbreak could work against this strategy as the growing number of cases would likely exceed the ability of the previous patients to provide donor sera as treatment.

### Potential future therapeutic strategies for COVID-19

As there is no approved treatment for COVID-19 till the present time, the researchers all over the world are working hard in order to find an effective treatment for this pandemic disease. In an attempt to participate in this battle, we proposed some new therapeutic approaches which could be used in the future in the fight against COVID-19, some of which are being studied as future treatment options for other viruses like influenza virus, SARS-CoV, MERS-CoV, and Ebola virus.

### Blocking the viral entry to the human cell

An interesting therapeutic strategy of blocking the viral entry to the human host cells was proposed by many researchers [55, 56]. Briefly, three proposed approaches would block the interaction between the S protein of SARS-CoV-2 and ACE2 receptors on the human cell surface thus, preventing the viral particles from entry which would subsequently prevent the infection. The first approach involves administration of the receptor-binding domain (RBD) of the S protein from SARS-CoV-2 virus which will bind to the ACE2 receptors leading to saturation of the available sites. In the second approach, an antibody would be administered against the ACE2 receptors to accomplish the same result of the first approach. A third approach would target the virion itself directly by using the ACE2 extracellular domain as a bait to bind to the S protein of SARS-CoV-19. Fusion of an Fc domain to ACE2 (ACE2-Fc) could allow prolonged circulation [55, 56].

### Small interfering RNA

Small interfering RNA (siRNA) is a class of double-stranded RNA molecules having length ranged from 20 to 25 base pairs. siRNAs have the ability to regulate the expression of certain genes, by a phenomenon known as RNA interference (RNAi). The siRNA-based therapeutic strategies have been developed and applied for antiviral, anticancer, and genetic diseases [4]. Some previous studies revealed that siRNA-based drugs were effectively utilized against SARS-CoV and MERS-CoV by using siRNAs targeting the sequences coding for the viral RNA-dependent RNA polymerase, helicase, protease, and the nucleoprotein N. Thus, this technology should be studied as a promising treatment strategy against COVID-19 to produce better therapeutic outcomes and to reduce the viral pandemic threat.

### Sphingosine mimics

Sphingosine 1-phosphate (S1P) is a lipid mediator which has diverse cellular activities. The sphingosine mimics are a group of immunosuppressants that can be used in certain infectious diseases. They can act as agonists of the sphingosine receptors leading to lymphopenia via sequestration of the lymphocytes in the lymph nodes causing immunosuppression. Recent studies have shown the therapeutic efficacy of using S1P in influenza-infected mice [57]. They noted that the intra-tracheal delivery of S1P agonist resulted in reduction in the lung injury and pro-inflammatory cytokine production. Thus, this approach of therapy could be used in the diminishing of the CRS that occurs in COVID-19 patients. Nevertheless, targeting the pro-inflammatory immune cells may not be a suitable line of treatment as it also affects the capacity of the host to clear the viral infection. Consequently, the use of S1P analogs should be utilized with caution in combination with antiviral drug in order to ensure effective clearance of the viral infection.

### Nuclear factor-kappa B inhibitors

Recently, it is found that the severity of SARS-Cov-2 lies partially in its ability to activate the nuclear factor-kappa B. Nuclear factor-kappa B (NF-kB) stimulates the expression of several genes which encodes the production of cytokines leading to the CRS that frequently occurs in patients with COVID-19 [58]. Furthermore, NF-kB expresses the platelet activator receptor which increases the likelihood of thrombi formation in the peripheral capillaries. Additionally, NF-kB results in the production of GTPase (specialized for the transport of RNA polymerase II into the nucleus) that plays a great role the transcription of mRNA of SARS-CoV-2 [59]. Consequently, in order to treat patients with COVID-19, we have to control the activity of NF-Kb by using NF-kB inhibitors like Amlexanox™.

### Cytokine receptors fc-fusion proteins

Recently, there are reports from Cambridge University which suggest that cytokine receptors Fc-fusion proteins can potentially serve as an antibody-like decoy to decrease the excessive levels of cytokines as a strategy of the treatment of COVID-19-infected patients [5, 60]. They utilized a new protein modification tool called QTY code, through which they can replace certain hydrophobic amino acids by other hydrophilic ones in particular cytokine receptors, including certain interleukin and interferon receptors. The QTY variant cytokine receptors display many physiological properties that are very similar to those of the native receptors without the presence of the hydrophobic segments. The receptors were then fused to the Fc region of IgG protein in order to form an antibody-like structure. These QTY code designs of the functional, water-soluble Fc-fusion as decoy therapeutic strategy to promptly remove the excessive cytokines in the hyperactive immune reactions that occur during CRS in COVID-19 seriously infected patients [60].

### Regulators of the intestinal microecology

Although the main symptoms of patients having COVID-19 are respiratory symptoms like fever, cough, and dyspnea, there are less common symptoms like the headache and some gastrointestinal symptoms such as diarrhea, nausea, and vomiting. Interestingly, it is observed that notable percentage of patients initially presented with those atypical gastrointestinal symptoms. As mentioned before, SARS-CoV-2 binds with ACE2 receptors which are highly abundant in the epithelia of both lungs and intestine of healthy individuals. Further analysis revealed that exposure of the epithelial cells of the small intestine to foreign pathogens significantly increased the expression of ACE2. Mutations in the ACE2 receptor may decrease expression of the antibacterial peptides in the cells of the intestine leading to changes in the intestinal microecology. Thus, researchers supposed that COVID-19 may have an effect on the intestinal flora by the ACE2 receptor [5]. Recent studies have presented that the use of intestinal tract microecological regulators (regulate the intestinal flora) can reduce the incidence of enteritis and respiratory-associated lung infection; thus, they can be used in the treatment of severe cases in order to maintain microbial balance in the intestine and to avoid the secondary bacterial infections [5]. Though, until now, there is no clinical evidence that the use of intestinal tract microecological regulators can have a role in the treatment of patients with COVID-19, it is still a potential treatment option, or may be used as an adjuvant therapy [61].

### Drugs targeting the host interactome of SARS-CoV-2

Targeting the host genes which are necessary for the viral growth and replication is called host interactome [62] which is an attractive new model of the treatment strategies for COVID-19. This approach relies on the theory that the short-term inhibition of these host functions in order to treat an acute viral infection would not have major adverse effects. Messina et al. [63] have developed a network-based model aiming to define the molecular aspects of pathogenic phenotype in SARS-CoV-2 infection. The resulting pattern could facilitate the structure-guided pharmaceutical and diagnostic research in order to identify potential new host targets. In addition, Cava et al. [64] reported that the incorporation of drug-gene interactions in the molecular docking analysis is very helpful in finding several drugs with antiviral activity which could be used alone or in combination with other therapeutic options as new therapeutic approaches in the battle against COVID-19 pandemic disease.

## Conclusion

Over the years, much research on coronaviruses has been conducted and produced various treatment strategies. Such results are likely to be applied to SARS-CoV-2 or any other evolving coronavirus in the future. With the continued hard efforts to prevent spread of SARS-CoV-2 globally, we hope that this pandemic disease will subside in a few months like SARS and MERS. Yet, this outbreak highlights the urgent need to design and produce new treatment strategies to fight against coronaviruses. Currently, our immediate action must be to achieve the infection control measures in order to prevent further worldwide transmission of COVID-19 and parallel conduction of clinical trials on the proposed therapeutic options.

### Future perspective

The increased number of people infected with SARS-CoV-2 all over the world and the associated increase in the mortality rate is an important public health issue. As the number of COVID-19 cases increase, the disease control become more difficult particularly that we have no effective drugs against COVID-19. In the current review, we presented some therapeutic strategies that could be used for the current and future treatment of SARS-CoV-2 infection. Nevertheless, further research and clinical studies would elucidate the significance of the findings of the current review.

## Data Availability

All data and material are available upon request.

## Abbreviations

COVID-19: Coronavirus disease 2019
SARS-CoV: Severe acute respiratory syndrome coronavirus
MERS-CoV: Middle East respiratory syndrome coronavirus
S protein: Spike protein
M protein: Membrane protein
E: Envelope
HE: Hemagglutinin-esterase
N: Nucleocapsid
ARDS: Acute respiratory distress syndrome
CRS: Cytokine release syndrome
VTE: Venous thromboembolism
JAK: Janus kinases
ACE: Angiotensin-converting enzyme
ARB: Angiotensin receptor blocker
CS: Cytokine storm
IL-1: Interleukin-1
IL-6: Iinterleukin-6
IL-8: Interleukin-8
TNF-α: Tumor necrosis factor-alpha
RBD: Receptor-binding domain
siRNA: Small interfering RNA
S1P: Sphingosine 1-phosphate
NF-kB: Nuclear factor-kappa B
